# Decreased expression of ADAMTS-1 in human breast tumors stimulates migration and invasion

**DOI:** 10.1186/1476-4598-12-2

**Published:** 2013-01-05

**Authors:** Vanessa M Freitas, Jônatas Bussador do Amaral, Thaiomara A Silva, Emerson S Santos, Flávia R Mangone, João de Jesus Pinheiro, Ruy G Jaeger, Maria A Nagai, Gláucia Maria Machado-Santelli

**Affiliations:** 1 Departamento de Biologia Celular e do Desenvolvimento, Instituto de Ciências Biomédicas, Universidade de São Paulo, Av. Prof. Lineu Prestes 1524, Ed Biomédicas 1 sala 428, São Paulo, SP, 05508-000, Brazil; 2 Departamento de Análises Clínicas, Faculdade de Ciências Farmacêuticas, Universidade de São Paulo Ribeirão Preto, Av. do Café, s/n, Ribeirão Preto, São Paulo, 14040-903, Brazil; 3 Disciplina de Oncologia, Departamento de Radiologia e Oncologia da Faculdade de Medicina da Universidade de São Paulo, Av. Dr. Arnaldo, 455, 4 andar, São Paulo, SP, 01246-903, Brazil; 4 Faculdade de Odontologia, Instituto de Ciências da Saúde, Programa de Pós-Graduação em Odontologia, Universidade Federal do Pará, Rua Augusto Corrêa, 01, Belém, Pará, 66075-110, Brazil

**Keywords:** ADAMTS-1, Breast cancer, Migration, Invasion, VEGF

## Abstract

**Background:**

ADAMTS-1 (a disintegrin and metalloprotease with thrombospondin motifs) is a member of the ADAMTS family of metalloproteases. Here, we investigated mRNA and protein levels of ADAMTS-1 in normal and neoplastic tissues using qPCR, immunohistochemistry and immunoblot analyses, and we addressed the role of ADAMTS-1 in regulating migration, invasion and invadopodia formation in breast tumor cell lines.

**Results:**

In a series of primary breast tumors, we observed variable levels of ADAMTS-1 mRNA expression but lower levels of ADAMTS-1 protein expression in human breast cancers as compared to normal tissue, with a striking decrease observed in high-malignancy cases (triple-negative for estrogen, progesterone and Her-2). This result prompted us to analyze the effect of ADAMTS-1 knockdown in breast cancer cells *in vitro*. MDA-MB-231 cells with depleted ADAMTS-1 expression demonstrated increased migration, invasion and invadopodia formation. The regulatory mechanisms underlying the effects of ADAMTS-1 may be related to VEGF, a growth factor involved in migration and invasion. MDA-MB-231 cells with depleted ADAMTS-1 showed increased VEGF concentrations in conditioned medium capable of inducing human endothelial cells (HUVEC) tubulogenesis. Furthermore, expression of the VEGF receptor (VEGFR2) was increased in MDA-MB-231 cells as compared to MCF7 cells. To further determine the relationship between ADAMTS-1 and VEGF regulating breast cancer cells, MDA-MB-231 cells with reduced expression of ADAMTS-1 were pretreated with a function-blocking antibody against VEGF and then tested in migration and invasion assays; both were partially rescued to control levels.

**Conclusions:**

ADAMTS-1 expression was decreased in human breast tumors, and ADAMTS-1 knockdown stimulated migration, invasion and invadopodia formation in breast cancer cells *in vitro*. Therefore, this series of experiments suggests that VEGF is involved in the effects mediated by ADAMTS-1 in breast cancer cells.

## Background

Extracellular matrix (ECM) components are involved in various aspects of tumor biology, including metastatic events. The ECM provides solid support to cells as well as a supply of cytokines and growth factors [1,2]. Cancer progression depends not only on the new abilities gained by neoplastic cells but also on the interaction between cells and their microenvironment [3]. ECM components are cleaved by proteases during physiological and pathological processes, and protease-induced breakdown of the ECM is essential for cancer cells to move through tissue barriers [4].

ADAMTS-1 (a disintegrin and metalloprotease with thrombospondin motifs) is a member of the ADAMTS family of metalloproteases. This secreted protease participates in various biological processes, such as inflammation, angiogenesis and development of urogenital system [5-8]. ADAMTS-1 has specific substrates that include modular proteoglycans, such as versican, aggrecan and brevican [9]. Despite the suggested roles for ADAMTS-1 in tumor invasion and metastasis, the effects of this molecule during cancer progression remain controversial. In 2008, Rocks et al. [10] showed that ADAMTS-1 contributes to tumor development by attracting fibroblasts and remodeling the extracellular matrix. Furthermore, some authors have shown that ADAMTS-1 is upregulated in pancreatic cancers with metastatic phenotypes [11]. On the other hand, decreased ADAMTS-1 expression has been described in human malignancies [12]. As a result, this protease was initially thought to inhibit angiogenesis in cancer and therefore act as an anti-cancer molecule [6] via the blockade of VEGFR2 phosphorylation by directly binding and sequestrating VEGF165 [7].

Here, we analyzed ADAMTS-1 mRNA expression in 60 human breast tumors, protein localization in 59 human samples and protein expression in 56 human samples, including normal and neoplastic tissues. To further evaluate the role of ADAMTS-1 in tumor biology, we studied the role of this protease in the regulation of migration and invasion in MDA-MB-231 and MCF7 breast cancer cells. Knocking down ADAMTS-1 expression using siRNA increased the migration and invasion of MDA-MB-231 cells. We also found a relationship between ADAMTS-1 and the activity of invadopodia, membrane protrusions related to the initial steps of cancer invasion. MDA-MB-231 cells with silenced ADAMTS-1 displayed increased VEGF expression in conditioned medium. Furthermore, this conditioned medium containing higher levels of VEGF induced HUVEC tubulogenesis. Our findings indicate that the effects of ADAMTS-1 in tumor invasiveness may be related to the availability of VEGF.

## Results

### ADAMTS-1 protein and mRNA expression in primary breast tumors

Quantitative real-time RT-PCR (qPCR) was used to analyze mRNA expression level of ADAMTS-1 in 60 primary breast tumors. ADAMTS-1 transcripts were expressed at varying levels (ranging from 0.1 to 7.3 AU) in the primary breast tumors analyzed. We determined a gene expression cut-off value of 0.7 (median value) that differentiated between ADAMTS-1 low expression and high expression in breast tumors. Using this cut-off, 39/60 (65%) of the breast tumors were classified as ADAMTS-1 low expressors, and 21/60 (35%) were classified as ADAMTS-1 high expressors. The clinicopathological characteristics of the patients are summarized in Table 1.

**Table 1:** Patients and tumor samples characteristics

**Variable**	**Categories**	***n***
Age (years old)	< 50	22 (36.7)
	≥ 50	38 (63.3)
Stage (TNM)	Stage I	2 (3.3)
	Stage II	25 (41.7)
	Stage III	26 (43.3)
	Stage IV	7 (11.7)
Tumor size (cm)	< 4.0	21 (35.0)
	≥ 4.0	39 (65.0)
Lymph node metastasis	No	20 (33.3)
	Yes	40 (66.7)
Estrogen receptor	Negative	27 (45.0)
	Positive	33 (55.0)
Progesterone receptor	Negative	33 (55.0)
	Positive	26 (43.3)
	Missing cases	1 (1.7)
ADAMTS-1	Negative	31 (51.7)
	Positive	29 (48.3)

Table 2 compares the mean ADAMTS-1 mRNA expression values in relation to the clinicopathological features of the 60 breast cancer patients.

**Table 2:** ADAMTS-1 expression (median value) according to clinicopathological features and biomolecular markers in primary breast tumors

**Variable**	**Categories**	***n***	**ADAMTS-1 expression**	***p *value**
Age (years old)	< 50	22	0.350	MW: 0.534
	≥ 50	38	0.475	
Stage (TNM)	Stage I	2	0.225	KW: 0.006
	Stage II	25	0.250	
	Stage III	26	0.625	
	Stage IV	7	1.100	
Tumor size (cm)	< 4.0	21	0.350	MW:0.950
	≥ 4.0	39	0.400	
Lymph node metastasis	No	20	0.450	MW: 0.712
	Yes	40	0.350	
Estrogen receptor	Negative	27	0.350	MW: 0.291
	Positive	33	0.600	
Progesterone receptor	Negative	33	0.550	MW: 0.731
	Positive	26	0.350	

KW: Kruskal Wallis test.

MW: Mann Whitney test.

**Figure 1:**
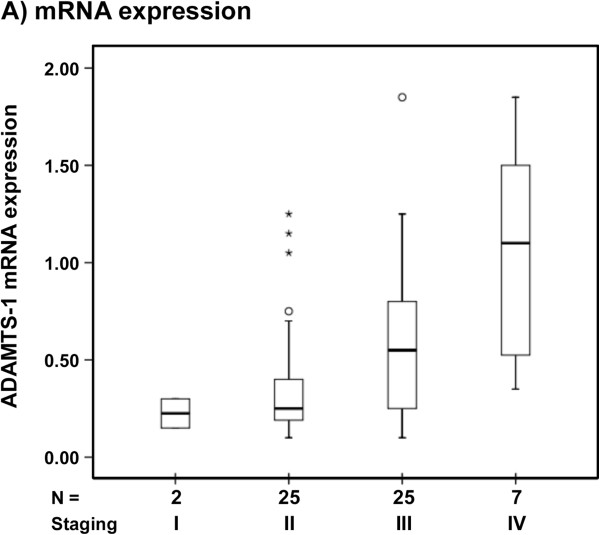
**The relative mRNA expression of ADAMTS-1 in primary breast tumors according to clinical stage (TNM).** The box plot shows data distributed in relation to the median values for ADAMTS-1 mRNA expression in 60 primary breast tumors stratified by clinical stage (p = 0.002). Three stage III cases with ADAMTS-1 expression above 2.0 (2.75, 4.95, 7.3) were excluded from the graphic for better visualization.

Statistical analyses demonstrated that high ADAMTS-1 mRNA expression was significantly associated with advanced clinical stages (p = 0.006) (Table 2 and Figure 1). However, there were no significant differences between ADAMTS-1 mRNA expression and age, tumor size, lymph node metastasis or the levels of estrogen or progesterone receptors. Neither overall survival (log-rank test; p = 0.459), nor disease-free survival (log-rank test; p = 0.481) were significantly different between patients with low or high ADAMTS-1 mRNA expression levels (data not shown).

**Figure 2:**
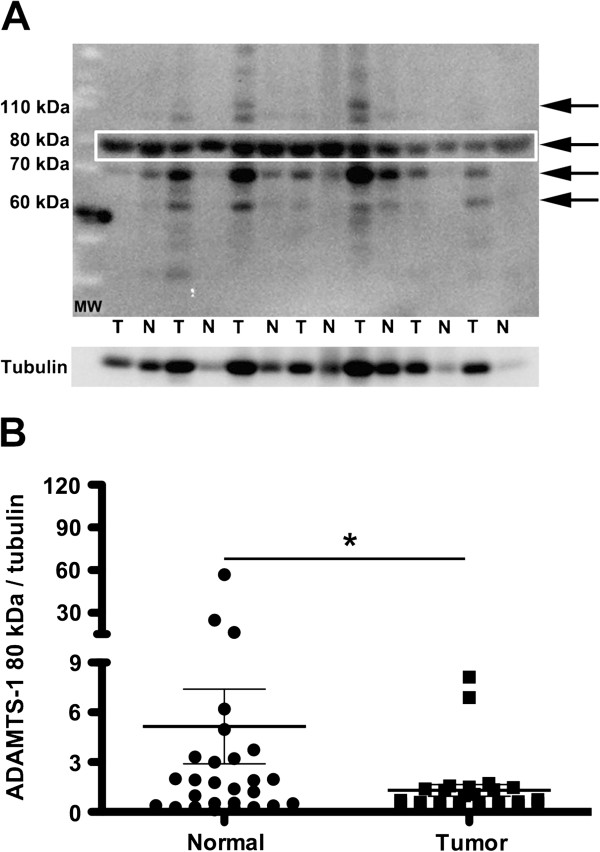
**Immunoblot of ADAMTS-1 in tumor (T) and adjacent normal tissue (N).** Four bands were identified as ADAMTS-1 (**A**, arrows). Ductal carcinomas and matched adjacent tissues were obtained from twenty-eight patient donors and analyzed. The 80 kDa band/tubulin ratio was significantly lower in tumors as compared to normal tissues (**B**). No differences were found among other bands obtained from neoplastic and normal tissues (not illustrated). The boxed area shows the 80 kDa bands that were evaluated by densitometry. The asterisks indicate significant data (p < 0.05).

Immunoblot analyses using an antibody raised against the N-terminus of ADAMTS-1 produced at least four different bands in samples from human patients, with approximate molecular weights of 110, 80, 70 and 60 kDa (Figure 2A). Moreover, the levels of ADAMTS-1 expression in tumors was lower compared to the expression levels in adjacent, normal tissues from the same patients, and this comparison was obtained by calculating the ratio between ADAMTS-1 and tubulin expression. The 80 kDa ADAMTS-1 form produced the strongest band, and its expression was lower in tumors as compared to normal adjacent tissues (Figure 2B). No differences were found among the other three bands when comparing the tumor samples to normal adjacent tissues (not illustrated).

**Figure 3:**
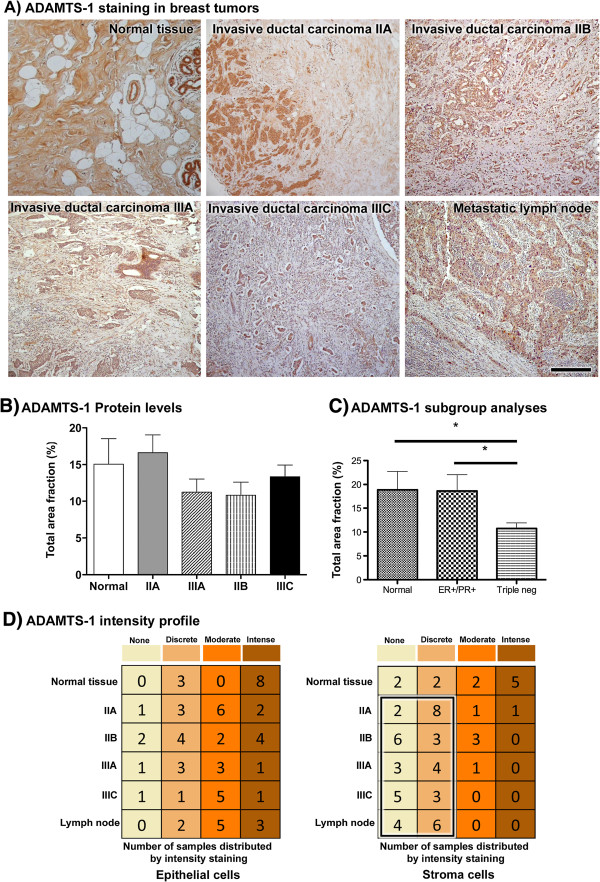
**ADAMTS-1 was more abundant in normal tissue stroma as compared to tumor stroma.** ADAMTS-1 staining is shown for normal tissue, invasive ductal carcinoma of grades IIA, IIB, IIIA and IIIC and metastatic lymph nodes (**A**). When the total area fraction was analyzed by immunohistochemistry, ADAMTS-1 showed no statistically significant difference in staining between normal and tumor tissues (**B**). However, a decreasing trend was observed in high-grade types (IIIA and IIB). Subgroup analysis revealed a significant decrease in ADAMTS-1 expression in triple negative tumors as compared to normal tissues and ER- or PR-positive tumors (**C**). The labeling intensity profile (**D**) shows that the expression of ADAMTS-1 in normal tissue was prominent in both epithelial cells and stroma. In contrast, ADAMTS-1 staining was discrete or absent in tumor stroma (**D**, boxed area), and this discrete or absent staining pattern was more evident in higher grades of malignancy (**D**, boxed area). Scale bar: 50 μm.

Immunohistochemistry (IHC) was carried out in samples from normal tissue; breast cancer tumors of grade IIA, IIB, IIIA and IIIC; and metastatic lymph nodes, and we evaluated ADAMTS-1 expression in the tumor cell cytoplasm and the surrounding stroma (Figure 3A). No significant differences were observed regarding the staining area fraction, even in the higher graded tumors (Figure 3B). However, high-grade tumors (IIIA and IIB) exhibited a trend towards decreased ADAMTS-1 expression in comparison to the lower tumor grades.

Next, we compared tumor and normal tissue subgroups, with tumor samples classified as either 1) estrogen (ER) and progesterone (PR) receptor positive tumors or 2) triple-negative tumors (ER-, PR-, Her-2). In triple-negative tumors, ADAMTS-1 protein expression was reduced in 40% of the cases as compared to both normal tissues and the ER or PR single-positive samples (Figure 3C).

We then assessed differences in staining intensity with regard to the ADAMTS-1 distribution among tumor cells or stroma. The labeling intensity profile (Figure 3D) showed that the expression of ADAMTS-1 in normal tissues was prominent in both epithelial cells and stroma. In contrast, ADAMTS-1 staining was discrete or absent in the tumor stroma, and this discrete or absent staining pattern was more evident in cases with higher grades of malignancy.

### Effects of ADAMTS-1 expression on cell migration

**Figure 4:**
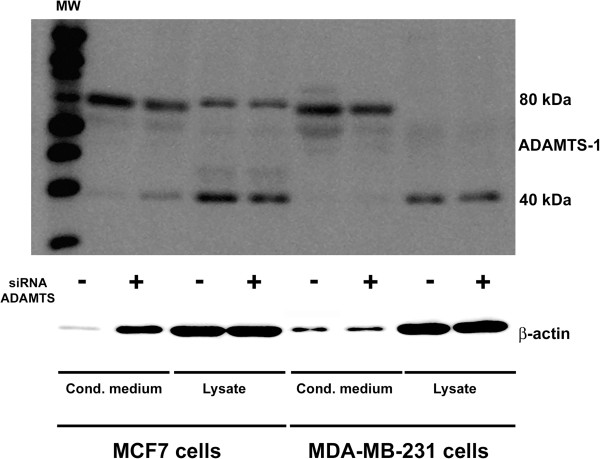
**ADAMTS-1 expression in cell lysates and conditioned medium from MCF7 and MDA-MB-231 cells.** These cells showed reduced expression of ADAMTS-1 following siRNA treatment. The lysates and conditioned media were analyzed by immunoblot, and an 80-kDa band was prevalent in the conditioned medium from both cells lines, but more prominently in MDA-MB-231. A 40-kDa smaller band was predominant in the cell lysates. The immunoblot also illustrated the efficiency of ADAMTS-1 siRNA knockdown. These experiments were carried out at least six times, and similar results were consistently obtained.

We also investigated ADAMTS-1 expression levels in breast tumor cell lines, such as MCF7 and MDA-MB-231. In these cells, siRNA treatment reduced the expression of ADAMTS-1. In addition, cell lysates and conditioned medium were analyzed by immunoblot, and an 80 kDa band was observed for the conditioned medium from both cell lines (Figure 4), whereas a 40 kDa band was present predominantly in the cell lysates. In addition, the immunoblot illustrated the efficiency of ADAMTS-1 siRNA knockdown (Figure 4).

**Figure 5:**
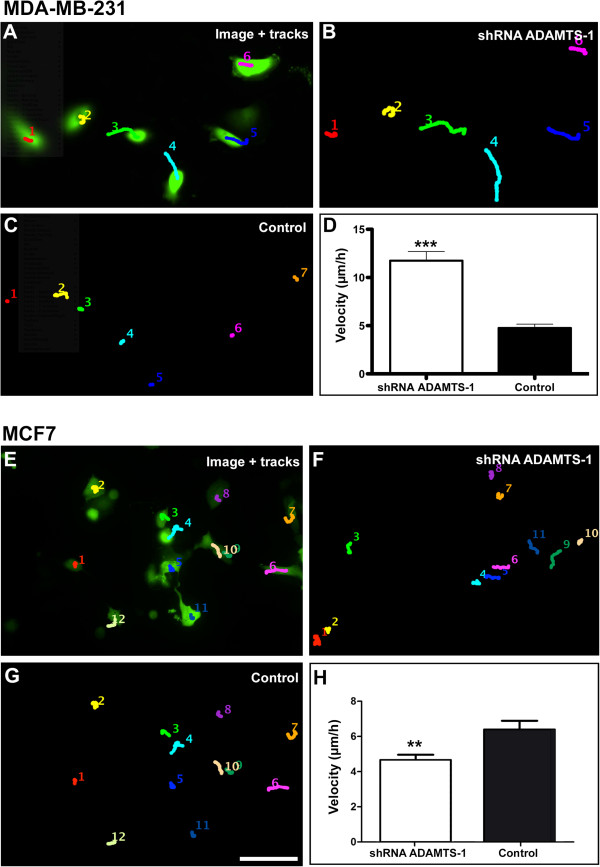
**ADAMTS-1 knockdown increased the velocity of MDA-MB-231 cells, as shown by time-lapse video microscopy.** The cells were transfected with either ADAMTS-1 shRNA-GFP (treated) or non-silencing shRNA-GFP (controls). The fluorescent images were superimposed with the MDA-MB-231 cell trajectories (**A**, color tracks) and are illustrated as manual markings on the same cell at different time points. The cells were recorded after 4.5 hours. The treated cells (**B**) demonstrated longer trajectories over time as compared to the controls (**C**). The velocity of the treated cells was also higher as compared to the controls (**D**). The asterisks indicate significant data in comparison to the controls (p < 0.001). The results in B represent the mean ± standard error obtained from 6 experiments. Scale bar: 100 μm. ADAMTS-1 knockdown decreased the velocity of MCF7 cells, as shown by time-lapse video microscopy. The cells were transfected with either ADAMTS-1 shRNA-GFP (treated) or non-silencing shRNA-GFP (controls). The fluorescent images were then superimposed with MCF7 cell trajectories (color tracks) and are illustrated as manual markings on the same cell at different time points (**E**). The cells were recorded after 4.5 hours. The treated cells (**F**) presented shorter trajectories over time as compared to the controls (**G**), and the velocity of the treated cells was lower in comparison to the controls (**H**). The asterisks indicate significant data in comparison to the controls (p < 0.01). The results in D represent the mean ± standard error obtained from 6 experiments. Scale bar: 100 μm.

Next, the effect of ADAMTS-1 on cell migration was analyzed using time-lapse imaging. For these experiments, we utilized shRNA-GFP. Cells were transfected with either ADAMTS-1 shRNA-GFP or control shRNA-GFP, and only fluorescent cells were analyzed. We measured the cell distance from the initial position during a set time interval, which yielded a mean velocity measurement (μm/hours). Functional ADAMTS-1 knockdown stimulated the migratory activity of MDA-MB-231 cells, and these cells presented a mean velocity of 11.74 ± 0.9603 μm/hour, which was at least 2 times higher than that of the controls (4.756 ± 0.3993 μm/hour) (Figure 5A-D).

MCF7 cells were used in migration experiments to address whether ADAMTS-1 knockdown stimulated migration in other cell lines, including this non-invasive breast adenocarcinoma cell line. The mean velocity of the control cells was 6.479 ± 0.4896 μm/hour, whereas the velocity of the treated cells was 4.676 ± 0.3026 μm/hour. Therefore, in MCF7 cells, migration was not stimulated and was instead reduced (Figure 5E-H).

### VEGF availability and VEGFR2 expression

**Figure 6:**
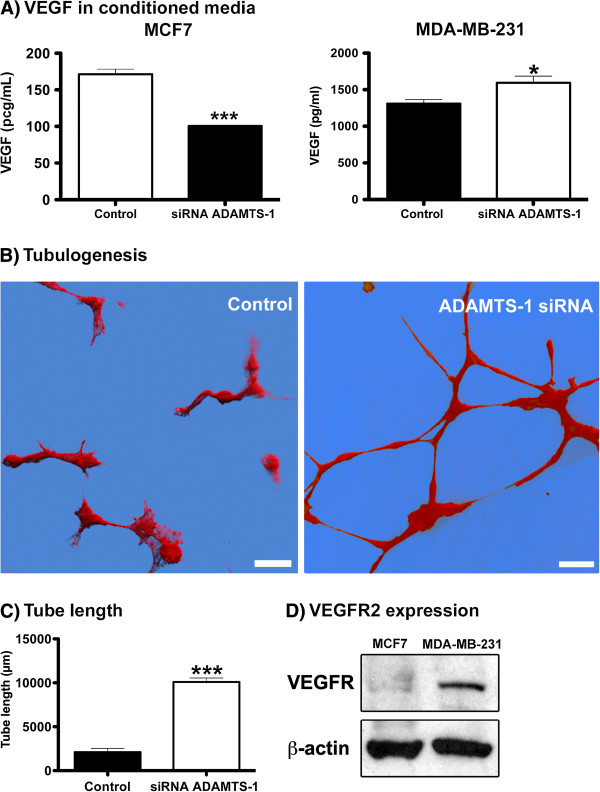
**Decreased ADAMTS-1 induced increased VEGF levels in conditioned medium from MDA-MB-231 cells (A) and tubulogenesis in HUVECs (B).** ELISAs were carried out to quantify the VEGF levels in conditioned medium from MDA-MB-231 or MCF7 cells treated with siRNA targeting ADAMTS-1. MDA-MB-231 cells with reduced ADAMTS-1 expression exhibited a statistically significant increase in VEGF in conditioned medium (**A**, right graph). Conversely, MCF7 cells with ADAMTS-1 silenced by siRNA showed a decrease in VEGF levels in conditioned medium (**A**, left graph). HUVECs were grown in conditioned medium derived from MDA-MB-231 cells transfected with ADAMTS-1 siRNA. In this situation, tubulogenesis was induced after 6 hours (B, right panel). The control cells (medium from cells transfected with control siRNA) showed no particular architecture (B, left panel), whereas tube length was increased in HUVECs treated with conditioned medium derived from MDA-MB-231 cells transfected with ADAMTS-1 siRNA (**C**). The immunoblot shows the difference in VEGFR expression between MDA-MB-231 and MCF7 cells (**D**). The asterisks indicate significant data in comparison to the control (*p < 0.05; ***p<0.001). The panels in (**B**) represent volumetric renderings from at least 10 confocal optical sections (software Imaris 7.1). The results in (**C**) represent the mean ± standard error of three experiments carried out at least three times. Scale bars: 50 μm.

As ADAMTS-1 knockdown had different effects in MCF7 and MDA-MB-231 cells, we next focused on the mechanisms of ADAMTS-1 that were responsible for these effects on cell migration. Previous reports have demonstrated that ADAMTS-1 sequesters VEGF [7]. Thus, we hypothesized that decreasing ADAMTS-1 could increase the availability of VEGF. ELISAs were performed to determine the relative VEGF concentrations in conditioned medium from either MDA-MB-231 or MCF7 cells that received ADAMTS-1 siRNA knockdown treatment. We found that MDA-MB-231 cells in conditioned medium exhibited a statistically significant increase in VEGF when ADAMTS-1 expression was reduced (Figure 6A). Conversely, MCF7 cells in conditioned medium showed a decrease in VEGF when ADAMTS-1 was silenced using siRNA (Figure 6A).

To address the functional role of decreased ADAMTS-1 levels in MDA-MB-231 cells and the consequent increase in VEGF, we carried out endothelial tubulogenesis assays.

In MDA-MB-231 cells, ADAMTS-1 expression was silenced by siRNA, and cells transfected with scrambled siRNA served as the controls. We obtained conditioned medium from treated and control cells, and we used this conditioned medium to induce tubulogenesis in human endothelial cells (HUVECs).

HUVECs were grown in conditioned medium derived from MDA-MB-231 cells transfected with ADAMTS-1 siRNA, which created a condition where endothelial cells could be cultured in media with reduced levels of ADAMTS-1. In this situation, tubular network formation was increased as compared to HUVECs grown with MDA-MB-231 control cells (Figure 6B). We next measured the length of the tubes formed in the tubulogenesis assays and determined that tube formation with conditioned medium from MDA-MB-231 cells and reduced ADAMTS-1 expression was increased by 4-fold as compared to the controls (Figure 6C).

Recent reports have also shown that VEGF is related to the migration of cancer cells [13-18]. Therefore, we hypothesized that the differences between MCF7 cell migration and MDA-MB-231 cell migration may be associated with VEGF expression levels in these cell lines. Thus, we performed immunoblots to compare VEGFR2 protein expression levels between MCF7 and MDA-MB-231 cells, and we found that VEGFR2 expression levels in MDA-MB-231 cells were 1.5-fold higher than in MCF7 cells (Figure 6D).

### VEGF and ADAMTS-1 in cell migration and invasion

**Figure 7:**
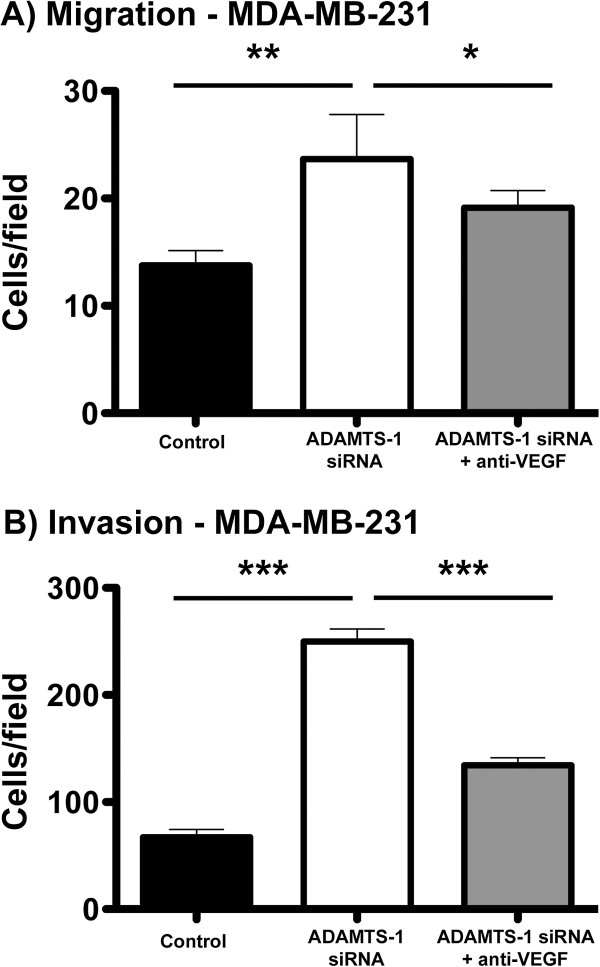
**Increased cell migration and invasion was stimulated by decreased expression of ADAMTS-1 and was partially rescued by a VEGF functional blocking antibody (A, B).** ADAMTS-1 knockdown increased the migration and invasion of MDA-MB-231 cells. The migration of MDA-MB-231 cells with ADAMTS-1 silenced by siRNA increased two-fold as compared to the control. The addition of an anti-VEGF blocking antibody partially rescued this migration to control levels (**A**). Invasion assays were carried out in Boyden chambers coated with Matrigel (**B**). Cells with silenced ADAMTS-1 demonstrated three-fold increased levels of invasion as compared to the controls. Invasion was partially rescued to control levels in cells pretreated with the VEGF blocking antibody. The asterisks indicate significant data (*p < 0.05, **p < 0.01, ***p < 0.001). The results represent the mean ± standard error of three experiments carried out at least three times.

To analyze the putative role of VEGF during the cell migration of ADAMTS-1 siRNA-treated cells, we carried out cell migration and invasion assays. In these assays, a VEGF blocking antibody was included. We found that decreasing ADAMTS-1 levels enhanced both the migration and invasion of MDA-MB-231 cells. Furthermore, treatment with a VEGF blocking antibody partially rescued both cell migration and invasion to the levels obtained with the control (Figure 7A-B).

**Figure 8:**
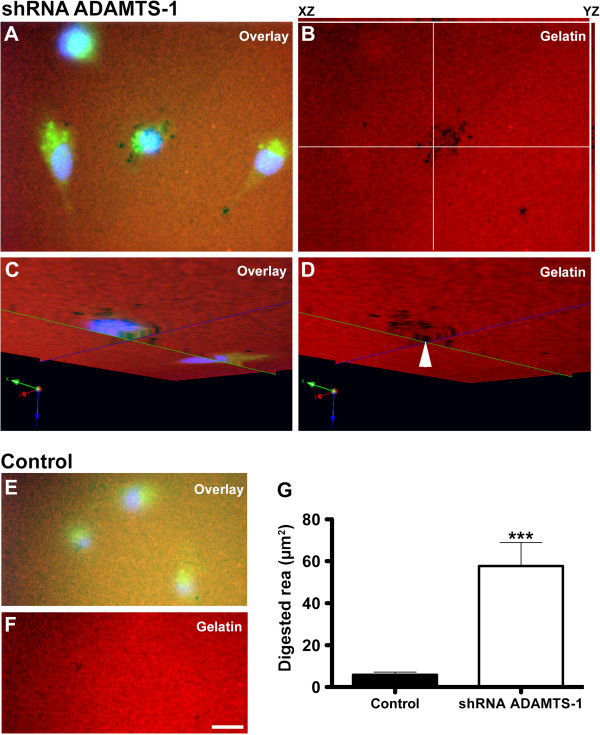
**ADAMTS-1 knockdown increased invadopodia formation in MDA-MB-231 cells.** Cells were grown on gelatin-Alexa-567 for 6 hours. Image **A** is a merged image of the blue (nuclei stained with DAPI), red (gelatin-Alexa 567) and green (ADAMTS-1 shRNA-GFP) channels. Z-projections of 10 optical sections together with orthogonal projections onto XZ (top) and YZ (left) planes were generated (**B**). The white lines in (**B**) indicate points of the XY image projected to generate orthogonal (perpendicular) XZ (top) and YZ (right) planes. Orthogonal projections of XZ and YZ illustrate digestion at the ventral surfaces of cells. Substrate digestion could be clearly observed when planes XY, XZ and YZ were viewed in the same volume (**C** and **D**, arrowhead). Images C and D were rotated to observe the cell’s ventral surface. The control samples (treated with non-silencing shRNA-GFP) show discrete matrix digestion (**E** and **F**). The area measurements demonstrate that ADAMTS-1 knockdown significantly increased matrix digestion (**G**). Digested areas in close proximity to the cell ventral surfaces indicate that ADAMTS-1 knockdown induced invadopodia activity. The asterisks indicate significant data in comparison to the control (*p < 0.05, ***p < 0.001). A total of 10 random fields were imaged per experimental group. Scale bar: 10 μm.

Furthermore, ADAMTS-1 knockdown increased invadopodia activity in comparison to control siRNA-treated cells, as shown using a fluorescent gelatin assay (Figure 8A-F). The digested gelatin areas (black spots in the fluorescent background) were measured using Image J, and MDA-MB-231 cells treated with ADAMTS-1 siRNAs exhibited at least a 5-fold increase in digested areas as compared to the controls.

## Discussion

ADAMTS-1 expression decreased in human breast tumors *in vivo*, mainly in triple negative cases (ER-, PR-, and Her-2), and ADAMTS-1 knockdown was shown to stimulate migration, invasion and invadopodia formation in breast cancer cells *in vitro*. Our series of experiments further suggest that VEGF is involved in these effects of ADAMTS-1 in breast cancer cells. To our knowledge, this is the first report establishing this relationship in human breast cancer cells.

ADAMTS-1 (a disintegrin and metalloproteinase with thrombospondin motifs) was the first described isoform in this family [19], and this identification was based on its elevated expression in cachexia-inducing adenocarcinomas in mice. Its multidomain structure links this secreted protein to various cellular functions; for example, it is a potent inhibitor of angiogenesis and plays an important role in follicular rupture and ovulation [20] and urogenital development, as demonstrated by the characteristics of knockout animal models [21].

Altered ADAMTS-1 expression has been reported in different types of tumors, including breast cancer [11,12]. However, the role of ADAMTS-1 in human breast cancer is not fully understood and requires further investigation. Lu et al. (2009) [22] reported that ADAMTS-1 was overexpressed in 39.7% of breast tumors, and these authors further demonstrated that ADAMTS-1 overexpression was associated with an increased risk of bone metastasis. Similarly, we observed variable levels of ADAMTS-1 mRNA expression in a series of primary breast tumors. However, the immunolocalization of ADAMTS-1 showed that the expression of this molecule was lower in triple negative tumors as compared to normal tissues. In this study, we compared the distribution of ADAMTS-1 in tumors of different clinical stages, which may explain the apparent discrepancies between our findings and previous data. The previous study by Lu et al. analyzed an array of breast cancer tissues without any consideration for the stage of the tumor. Therefore, we considered tumor stage in our analysis and also evaluated the expression of ADAMTS-1 in the tumor stroma, which was reduced in higher-staged tumors.

Immunoblots comparing normal tissue and cancer tissue revealed that ADAMTS-1 could be detected in four different bands: the 110 kDa band, which likely represents total protein; the 80 kDa, which may characterize the protease without the pro-domain or the activated form; and two smaller bands, which may correspond to activated ADAMTS-1 with additional proteolytic processing [23,24]. We also observed that the 80 kDa ADAMTS-1 band was decreased in breast tumors as compared to adjacent normal tissue samples. Thus, a reduction in ADAMTS-1, as determined by immunohistochemistry, may represent a decrease in the 80 kDa ADAMTS-1 band. With regard to the *in vitro* expression of ADAMTS-1, both the MCF7 and MDA-MB-231 cell lines exhibited a prominent ADAMTS-1 80 kDa band in conditioned medium.

Our results showed that MDA-MB-231 cells with reduced ADAMTS-1 expression demonstrated increased migration, velocity and invasion. In cancer progression, cell-to-cell detachment from the primary tumor and the acquisition of a motile phenotype are required for cells to become invasive and colonize distant organs, thereby producing a metastasis [25]. The spread of cancer cells to distant sites in the body is the major cause of death for cancer patients [26,27], and one major challenge in cancer therapy is to inhibit the spread of tumor cells from the primary tumor site to distant organs [28].

Previous reports have acknowledged the role of ADAMTS-1 in cell migration. Krampert et al. (2005) [29] studied the role of ADAMTS-1 in the healing of skin wounds. In this model, they observed that ADAMTS-1 played different roles in fibroblast migration depending on the concentration; a decrease in the level of this protein stimulated cell migration via the proteolytic activity of ADAMTS-1.

The effects of ADAMTS-1 knockdown on cell migration and invasion seem to be related to VEGF, as MDA-MB-231 cells with reduced ADAMTS-1 expression showed increased levels of VEGF in conditioned medium. The relationship between VEGF and ADAMTS-1 was recently reported, and the carboxyl-terminal domain of ADAMTS-1 was shown to be responsible for binding and sequestering VEGF [7]. This sequestration of VEGF by ADAMTS likely inhibits various functions of VEGF, such as its role in cell migration and invasion.

It has been described that ADAMTS-1 sequesters VEGF [7]. VEGF is known to enhance migration and invasion [13,15,16,18]. We then carried out combined multi-tiered experiments to relate the role of ADAMTS and VEGF during cell migration and invasion. ADAMTS-1 knockdown in MDA-MB-231 cells resulted in a decrease in ADAMTS-1 protease activity in conditioned medium. Furthermore, cells with reduced ADAMTS-1 expression demonstrated increased levels of VEGF in conditioned medium. Taken together, these results suggest that ADAMTS-1 knockdown decreased the presence of this protease in the conditioned medium of MDA-MB-231 cells, thus preventing the sequestration of VEGF and rendering this growth factor available to exert its cellular effects, including migration and angiogenesis [13,15,16,18].

To analyze the putative role of VEGF in the cell migration of MDA-MB-231 cells, we carried out migration and invasion assays in MDA-MB-231 cells with reduced ADAMTS-1 expression. To assess the effect of VEGF, we used a VEGF blocking antibody and found that ADAMTS-1 knockdown increased migration and invasion, as expected. However, treatment with blocking antibodies partially rescued both cell migration and invasion, and these results suggest a close relationship between ADAMTS-1 and VEGF in regulating cell migration and invasion.

The evidence presented here establishes a relationship between ADAMTS-1 and VEGF, and our results also indicated that VEGF in conditioned medium from MDA-MB-231 cells with ADAMTS-1 silenced initiated tubulogenesis in HUVEC cells. ADAMTS-1 has been described as a protease with angioinhibitory properties [6] that significantly blocks VEGFR2 phosphorylation and suppresses endothelial cell proliferation. In addition, the inhibition of ADAMTS-1-related angiogenesis is related to the sequestering of VEGF.

VEGF also induces invadopodia formation by increasing the activity of MMP-2, MMP-9 and MT1-MMP [15]. MDA-MB-231 cells with ADAMTS-1 knockdown demonstrated increased invasion in Boyden chambers, and cells with reduced ADAMTS-1 expression also demonstrated increased invadopodia formation. Therefore, MDA-MB-231 cells with depleted levels of ADAMTS-1 may increase the availability of VEGF, which could enhance invadopodia formation and/or activity.

Various authors have demonstrated invadopodia formation and/or activity in MDA-MB-231 cells using a variety of approaches. For example, invadopodia activity in MDA-MB-231 cells has been reported in src-transformed cells [30,31], in cells cultured on fibronectin [32] and in cells treated with growth factors [33]. Most of these studies were carried out in cells grown for at least 16 hours; in contrast, our results revealed invadopodia activity over a short timeframe as well as increased matrix digestion in MDA-MB-231 cells when ADAMTS-1 is knocked down.

Cancer cells rely on invadopodia to initiate invasive activity [30,34], as these formations are enriched with actin filaments (F-actin) and components needed for actin assembly, including neural-Wiskott Aldrich Syndrome protein (N-WASP) and cortactin [30,35-37]. Therefore, it was not surprising that the functional knockdown of ADAMTS-1 stimulated the migratory and invasive activity of MDA-MB-231 cells. On the other hand, ADAMTS-1 knockdown had the opposite effect on MCF7 cells and reduced their migratory activity. This discrepancy could be the result of different biological behaviors between these cell lines. It is well known that MCF7 cells express estrogen and progesterone receptors, while MDA-MB-231 cells do not. Thus, MDA-MB-231 cells may be more aggressive and invasive as compared to MCF7 cells.

Another possible explanation for these differences in migration and invasion could be related to VEGF and VEGF receptor levels. VEGFR2 expression levels in MDA-MB-231 cells were 1.5-fold higher in comparison to MCF7 cells, whereas MDA-MB-231 cells with reduced ADAMTS-1 expression showed augmented VEGF levels in the conditioned medium as compared to the controls. Taken together, these results suggest that MDA-MB-231 cells possess suitable machinery to interact with VEGF, a growth factor important for migration and invasion [13,15-18].

## Conclusions

**Figure 9:**
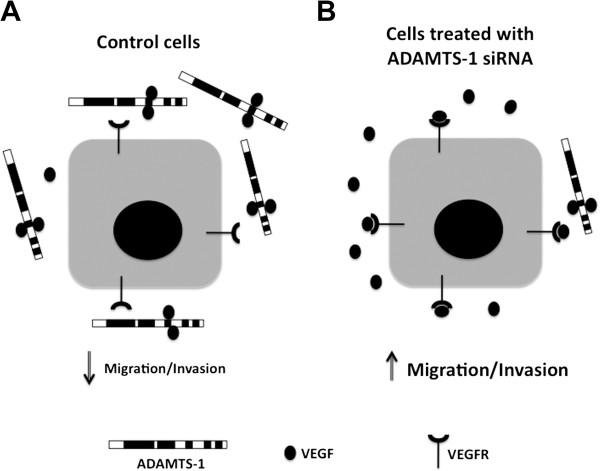
**Schematic diagram summarizing our current understanding of the role of ADAMTS-1 in breast cancer cells.** In normal cells (**A**), the extracellular environment is enriched for ADAMTS-1. This protease sequesters VEGF, thus preventing the effects of this growth factor on migration and invasion. In contrast, tumor cells (**B**) exhibit lower levels of ADAMTS-1 in the extracellular matrix. In this scenario, VEGF is no longer sequestered and becomes freely available to bind VEGFR, thereby increasing the migration and invasion of breast cancer cells.

Our study was the first to describe lower levels of ADAMTS-1 protein in triple-negative breast cancer cases (ER-, PR-, and Her-2), and our immunoblot analyses indicated that ADAMTS-1 expression was reduced in tumors in comparison to contralateral normal tissues. In addition, ADAMTS-1 knockdown stimulated migration, invasion and invadopodia formation in breast cancer cells *in vitro*. This series of experiments suggested that VEGF is involved in ADAMTS-1 effects on breast cancer cells; the diagram depicted in Figure 9 illustrates our current understanding of ADAMTS-1 in breast cancer. In normal cells, the extracellular environment is enriched with ADAMTS-1, and this protease sequesters VEGF, thus preventing the effects of this growth factor on migration and invasion. On the other hand, breast cancer cells exhibit lower levels of ADAMTS-1 in the extracellular matrix, and in this scenario, VEGF is no longer sequestered and becomes freely available to bind VEGFR, which increases breast cancer cell migration and invasion. Future investigations are needed to determine the precise mechanisms by which ADAMTS-1 and VEGF regulate the invasiveness of cancer cells.

## Material and methods

### Tissue samples, patient characteristics, RNA extraction and quantitative real-time PCR (qPCR)

Sixty tumor samples and 20 normal tissues adjacent to the tumors were obtained from 60 breast cancer patients at the Hospital do Câncer, A. C. Camargo, São Paulo, Brazil. Patient age ranged from 23 to 85 years (median 54 years). Tumor samples were dissected to remove any residual normal tissue before freezing and storing the samples in liquid nitrogen. The largest diameter of the tumors was recorded. Microscopic examinations were performed to determine the average number of lymph node metastases in 24 patients. Tumor metastasis into the lymph nodes was detected in 40 patients. Histopathological review of all the tumor slides was performed to confirm the diagnosis. All tumors were classified according to the WHO Histological Typing of Breast Tumors. Infiltrating ductal carcinomas were studied, and the clinical stage was assigned based on the UICC TNM (tumor, nodes, metastases) staging system.

The Institutional Ethics Committee approved this study, and all subjects provided informed consent.

Tissue specimens were pulverized under liquid nitrogen using a Frozen Tissue Pulverizer (Thermovac Industries Corporation, Copiague, NY, USA), and total RNA was extracted using the acid guanidinium thiocyanate-phenol-chloroform method [38]. Ten micrograms of total RNA, previously treated with DNaseI, was reverse transcribed using a High Capacity cDNA Archive Kit (Applied Biosystems, Carlsbad, CA, USA). qPCR was performed using an Applied Biosystems 7500 Real-Time PCR System, and each cDNA sample was analyzed in duplicate. The PCR reactions were carried out in a total volume of 25 μl according to the manufacturer’s instructions for the SYBR Green PCR Core reagent (PE Applied Biosystems). The following PCR primers were used: *ADAMTS*-*1* forward, 5’- TGTGGTGTTTGCGGGGGAAATG-3’ and reverse, 5’- TCGATGTTGGTGGCTCCAGTT -3’; and glyceraldehyde-3-phosphate dehydrogenase (*GAPDH*) forward, 5’-CCTCCAAAATCAAGTGGGGCG-3’ and reverse, 5’-GGGGCAGAGATGATGACCCTT-3’. The algorithm geNorm was used to define the calibrator gene among the RLP13, HPRT, ACTB and GAPDH transcripts. As a result, the relative gene expression was normalized, with GAPDH expression serving as the internal control. The average value from two groups of 10 normal tissue samples served as a reference sample. The results were expressed as the *n*-fold difference in target gene expression relative to the expression of the GAPDH gene and the reference sample. The relative expression was calculated using the 2^-ΔΔCT^ method (CT = fluorescence threshold value; ΔCT = CT of the target gene - CT of the reference gene (GADPH); ΔΔCT = ΔCT of the tumor sample - ΔCT of the reference sample).

### Immunohistochemical analysis

Tissue microarray slides from normal human and breast cancer samples were obtained from Imgenex (San Diego, CA; IMH-364). Fifty-nine samples in 4-μm sections were analyzed, including 35 cases of invasive ductal carcinoma (IDC), 1 case of sarcomatoid carcinoma, 1 case of intraductal papillary carcinoma, 1 case of atypical medullary carcinoma, 1 case of metaplastic carcinoma, 1 case of ductal carcinoma *in situ*, 10 cases of cancer metastasis and 9 samples of normal breast tissue adjacent to cancer tissue. Among these samples, 19 were triple-negative tumors, with 7 ER- and PR-positive cases. For antigen retrieval, 10 mM of citrate buffer (pH 6.0) with 0.05% Tween 20 was applied for 30 minutes at 100°C. Then, the EnVision method (EnVision; Dako Corp., Carpinteria, CA, USA) was used to analyze the TMAs, and diaminobenzidine served as the chromogen.

ImageJ public domain software (http://rsb.info.nih.gov/ij/) was used for image analysis. The DAB channel was separated by the color deconvolution plugin. The stained areas were segmented and measured. Quantification involved either DAB labeling divided by hematoxylin staining or scoring the labeled intensity of tumor cells or stroma. Immunohistochemical staining was quantitatively assessed by three independent observers (VMF, JBA, RGJ) with minimal interobserver variability (<5%).

### Cell lines and transfection

MCF7 and MDA-MB-231 cells were cultured in Dulbecco’s Modified Eagle’s Medium-F12 (DMEM-F12, Sigma) supplemented with 10% fetal bovine serum (FBS; Cultilab, Campinas, SP, Brazil). Human umbilical vascular endothelial cells (HUVECs) were cultured in 199 Medium supplemented with LSGS (Gibco). The cells were maintained in 25 cm^2^ flasks in a humidified atmosphere of 5% CO_2_ at 37°C.

MCF7 and MDA-MB-231 cells were transfected with commercially available siRNA targeting ADAMTS-1 (Santa Cruz Biotechnology Inc., Santa Cruz, CA, USA), according to the manufacturer’s instructions. One day prior to transfection, subconfluent MCF7 and MDA-MB-231 cells were cultured in DMEM supplemented with 10% FBS without antibiotic-antimycotic solution. The cells were incubated with a complex formed by the siRNA (50 nM), transfection reagent (Lipofectamine 2000, Invitrogen) and transfection medium (Opti-MEM I, Invitrogen) for 30 h at 37°C.

SureSilencing shRNA plasmids for human ADAMTS-1 with GFP (#KH01149G, SABiosciences, Frederick, MD, USA) were used for the time-lapse experiments.

Cells transfected with scrambled siRNA or shRNA served as controls. These assays were performed in triplicate.

### Time-lapse fluorescence microscopy

For the time-lapse fluorescence microscopy, shRNA-GFP was used to silence ADAMTS-1, as previously described. Cells transfected with scrambled interference RNA served as control. The migration was determined using a time-lapse fluorescence microscope with an Olympus IX 81 inverted microscope equipped with an Orca R2 CCD camera. Video recordings were conducted using Cell Observer image software (Olympus). Treated and control cells were maintained at 37°C in a temperature-controlled chamber, and images were collected every 5 minutes (over a total of 4 hours and 30 minutes). MTrack J plugin (Image J software) was used to measure cell velocity.

### Migration and invasion assays

Transwell inserts (with 8 μm pores) in 12-well plates (BD Biosciences) were used for the migration and invasion assays, where ADAMTS-1 was silenced by siRNA (Santa Cruz).

In the migration assays, treated and control cells (10^5^) were plated into the upper chamber containing 1 mL of DMEM-F12 without serum. The lower chamber was filled with 1.5 mL of DMEM-F12. After 24 hours in culture, the cells were fixed with 4% paraformaldehyde and post-fixed with 0.2% crystal violet in 20% methanol. Cells on the upper side of the filter were removed with a cotton swab. The migrating cells on the lower side of the filter were photographed and counted. These experiments were performed in triplicate and were repeated at least three times.

For the invasion assays, the filters were coated with 10 μl of Matrigel (10-13 mg/ml). Treated and control cells (10^5^) were plated into the upper chamber containing 1 mL of DMEM-F12 without serum. The lower chamber was filled with 1.5 mL of DMEM-F12. After 48 hours in culture, the cells were fixed and stained. The cells on the upper side of filter were removed, as described above, whereas the invading cells on the lower side of the filter were photographed and counted. These experiments were performed in triplicate and were repeated at least three times.

To determine the role of VEGF in migration and invasion following ADAMTS-1 depletion, cells with silenced ADAMTS-1 (10^5^) and controls were plated into the upper chamber containing 1 mL of DMEM-F12 without serum. The cells were incubated with anti-VEGF blocking antibody (1 μg/ml R&D Systems) or non-specific mouse IgG (1 μg/ml Millipore). The lower chamber was filled with 1.5 mL of DMEM-F12 supplemented with 10% of serum. The migration and invasion assays were conducted as previously described.

### ELISA

The level of VEGF in conditioned medium was quantified using MCF7 and MDA-MB-231 cells and the Human VEGF ELISA kit (Invitrogen). Cells were transfected with either ADAMTS-1 siRNA or control siRNA, followed by serum starvation for 24 h. The conditioned media were collected and centrifuged at 1,000 rpm for 5 min at 4°C to remove cellular debris. The conditioned medium was then analyzed by ELISA according to the manufacturer’s instructions, and the results were expressed in pg/mL.

### Tubulogenesis assay

ADAMTS-1 expression was silenced by siRNA in MDA-MB-231 cells, and cells that were transfected with scrambled siRNAs served as controls. We obtained conditioned medium from treated and control cells, as previously described, and this conditioned medium was used to induce tubulogenesis in human endothelial cells (HUVECs).

Three-dimensional HUVEC cultures were seeded on a solidified layer of reduced growth factor (RGF) Matrigel, approximately 1-2 mm in thickness. A round 13-mm coverslip coated with 20 μl of RGF-Matrigel (Trevigen, Gaithersburg, MD, kindly provided by Dr. Matthew Hoffman, NIDCR, NIH) was placed in each well of a 24-well plate. Then, a 100-μl drop of the cell suspension (3.5 × 10^5^ cells/ml) was placed on top of the Matrigel and incubated in HUVEC complete medium. After two hours, the non-adherent cells were washed, and the complete medium was replaced with serum-free medium and conditioned for 24 hours with confluent two-dimensional cultures of either control or siRNA-treated MDA-MB-231 cells. The HUVEC cells remained in these conditions for 4 hours, followed by fixation in 4% paraformaldehyde in PBS, permeabilization with 0.5% Triton X-100, actin staining with rhodamine-phalloidin (Invitrogen-Molecular Probes, Eugene, OR) and mounting with ProLong-DAPI (Invitrogen). The images were acquired using a Zeiss LSM 510 laser scanning confocal microscope (Carl Zeiss, Oberkochen, Germany) and analyzed using Imaris 7.1 (Bitplane Inc, South Windsor, CT, USA) and Wimasis (Wimasis GmbH, Munich, Germany).

### Fluorescent substrate degradation assay

To assess the role of ADAMTS-1 in MDA-MB-231 cell invadopodia formation, we carried out a fluorescent gelatin substrate degradation assay. The substrate was prepared using gelatin conjugated to Alexa 568 (Invitrogen, Eugene, OR, USA), and the conjugation followed the manufacturer’s instructions. MDA-MB-231 cells (2 × 10^4^/mL) that were transfected with ADAMTS-1 shRNA were plated onto the fluorescent gelatin substrate and incubated in DMEM with 10% FBS at 37°C for 6 hours. The control cells were treated with scrambled non-silencing shRNA. Treated and control cells were fixed in 4% paraformaldehyde in PBS and mounted using ProLong containing DAPI (Invitrogen).

The images were obtained with an Axiophot widefield fluorescence microscope using a 63x PlanApo 1.4 NA objective (Carl Zeiss) and acquired using a digital CCD monochromatic camera (CoolSnap HQ2, Photometrics Inc, Tucson, AZ, USA). To assess matrix digestion spots in the fluorescent substrate, at least ten Z sections per sample field were obtained using a piezoelectric device (PIFOC, Physik Instrumente, Germany) coupled to the objective. The microscope and devices were controlled using the Metamorph Premier 7.6 software (Molecular Devices, Sunnyvale, CA, USA).

We also examined shRNA-GFP-transfected cells (green channel) while screening for superimposed digested areas (dark spots) in the fluorescent gelatin matrix (red channel). ImageJ was used to measure the degraded areas. The red channel (fluorescent gelatin) was separately processed using a threshold tool to calculate the digested area (μm^2^) per cell. Volocity software (PerkinElmer, Waltham, MA, USA) was used to determine the orthogonal projections and image restorations using deconvolution algorithms.

### Western blot

Western blots were carried out to compare ADAMTS-1 levels in MCF7 and MDA-MB-231 cell lysates and conditioned medium and to verify siRNA transfection efficiency. The cells were lysed in RIPA buffer (150 mM NaCl, 1.0% NP-40, 0.5% deoxycholate, 0.1% SDS, 50 mM Tris pH 8.0) containing a protease inhibitor cocktail (Sigma). After centrifugation (10,000 g) for 10 min at 4°C, the supernatants were recovered and quantified (BCA kit, Pierce). The samples were resuspended in Laemmli buffer containing 62.5 mM Tris–HCl pH 6.8, 2% sodium dodecyl sulphate (SDS), 10% glycerol, 5% mercaptoethanol and 0.001% bromophenol blue. The conditioned medium (1 mL) was then ethanol-precipitated, and equal amounts (30 μg) of the cell lysates were electrophoresed on 10% polyacrylamide gels. The proteins were transferred to a Hybond ECL nitrocellulose membrane (Amersham) and blocked in TBS with 5% non-fat milk overnight at 4°C. Following one wash in TBS with 0.05% Tween 20 (TBST), the membranes were probed with antibodies against ADAMTS-1 (1:1,000, Abcam 28284), VEGFR2 (1:500, Santa Cruz) and β-actin (1:2,000, Sigma). The ECL protocol was used to detect proteins on the membrane.

We also used OncoPair INSTA-Blot Breast Tissue membranes (IMB-130a, b, c and e; IMGENEX, San Diego, CA, USA). These ready-to-use PVDF membranes contain denatured protein lysates from ductal carcinomas and were matched with adjacent tissues obtained from seven donors. Each membrane was probed with ADAMTS-1 (Abcam 28284) and alpha-tubulin (Abcam 4074) and revealed using the ECL protocol.

### Statistical methods

The median values of ADAMTS-1 mRNA expression were in accordance with the clinical and pathological variables and were compared using the Kruskal-Wallis and Mann–Whitney tests. For survival analyses, ADAMTS-1 expression was classified as low (<0.7) or high (≥0.7) according to its median expression value. The overall survival and disease-free survival rates were calculated using the Kaplan-Meier method, and the curves were compared using the log-rank test. The overall survival and disease-free survival were calculated from the day of diagnosis to the date of death and to the date in which recurrence was detected, respectively. The significance level was set at 5% for all tests. The statistical analyses were performed using SPSS software 15.0 (SPSS Inc., Chicago, IL). The protein data analyses and statistical significance were obtained using the Wilcoxon matched test. For *in vitro* experiments, the data were analyzed using Graph Pad Prism 5 software (Graph Pad Software, Inc., San Diego, CA, USA). A Student’s *t* test or ANOVA was used to assess the differences.

## Competing interests

The authors declare that they have no competing interests.

## Authors’ contributions

VMF, MAN and GMMS designed this study. FRM and MAN carried out the qPCR analysis of ADAMTS-1 expression in normal and neoplastic tissues. VMF and TAS performed experiments, including immunoblotting in ready-to-use PVDF membranes and analyzed ADAMTS-1 in cell line lysates and conditioned medium (Western blot). VMF, JBA, JJVP and RGJ carried out immunohistochemistry on TMA and performed the data analysis. VMF and JBA performed the experiments with shRNA and the time-lapse migration assay. VMF performed siRNA transfection, migration and invasion assays in Boyden chambers. VMF, RGJ and ESS carried out the tubulogenesis and invadopodia assays. JBA and GMMS performed the confocal microscopy analysis of HUVECs. VMF, MAN and RGJ prepared the manuscript, performed statistical analyses of the data and contributed to discussions and interpretations of the results. All authors have read and approved the final manuscript.
